# Diversity and dynamics of bacteria at the *Chrysomya megacephala* pupal stage revealed by third-generation sequencing

**DOI:** 10.1038/s41598-022-06311-7

**Published:** 2022-02-07

**Authors:** Wang Xu, Yu Wang, Ying-hui Wang, Ya-nan Zhang, Jiang-feng Wang

**Affiliations:** School of Biology & Baslc Medical Sciences, Soochow University, Suzhou, China

**Keywords:** Ecology, Microbiology

## Abstract

Characterization of the microbial community is essential for understanding the symbiotic relationships between microbes and host insects. *Chrysomya megacephala* is a vital resource, a forensic insect, a pollinator, and a vector for enteric bacteria, protozoa, helminths, and viruses. However, research on its microbial community is incomprehensive, particularly at the pupal stage, which comprises approximately half of the entire larval development stage and is important entomological evidence in forensic medicine. For the first time, this study investigated the bacterial communities of *C. megacephala* pupae at different ages using third-generation sequencing technology. The results showed that *C. megacephala* has a diverse and dynamic bacterial community. Cluster analysis at ≥ 97% similarity produced 154 operational taxonomic units (OTUs) that belonged to 10 different phyla and were distributed into 15 classes, 28 orders, 50 families, 88 genera, and 130 species. Overall, the number of bacterial OTUs increased with the development of pupae, and the relative abundance of *Wolbachia* in the Day5 group was significantly lower than that in the other groups. Within the pupal stage, Proteobacteria, Firmicutes, and Bacteroidetes were the dominant phyla of bacteria. At the genus level, *Wolbachia* and *Ignatzschineria* coexisted, a rarely known feature. In addition, we found *Erysipelothrix rhusiopathiae*, the etiological agent of swine erysipelas, which is rarely identified in insects. This study enriches the understanding of the microbial community of *C. megacephala* and provides a reference for better utilization and control of *C. megacephala.*

## Introduction

Insects are animals with the largest population worldwide. They are adaptable to various environmental and dietary conditions, a trait closely related to insect-associated microorganisms. Microorganisms exist in insects cells, particularly exoskeletons and intestines, and significantly impact aspects of insect biology, including pesticide resistance^1^, nutrition and digestion^2,3^, and immune defense^4,5^.

Studies of insect-associated microorganisms elucidate insect physiology and guide new methods for insect biological prevention and control. The premise of implementing biological prevention and control involves revealing the community structure and function of insect-associated microorganisms. The microbial community of insects varies with developmental stage, yet most studies only select specific time points within each developmental stage of insects and generated inconclusive results. In addition, only a few studies explored the dynamics of insect-associated microorganisms at the larval stage^6^. However, studies on the dynamics of insect-associated microorganisms in the pupal stage are lacking.

The rapid development of sequencing technology has shifted attention to the composition and function of insect-associated microorganisms. The traditional identification of microbial species requires isolation and culturing of microorganisms. However, most microorganisms cannot be cultured and remain uncharacterized with their species underestimated. In recent years, microbial-related research is widely using high-throughput next-generation sequencing (NGS) technologies, such as MiSeq (Illumina) and 454 (Roche) sequencing. However, the NGS platform generates relatively short sequences, which limits the data quality. The more recent third-generation sequencing technology improves the accuracy of analyzing microbial communities. The third-generation technology generates long reads, over 10,000 bp using the single-molecule real-time (SMRT) method, with a < 1% average error rate post-annealing with the SMRTbell adapter^7^. Few studies to date have employed the third-generation sequencing technology to study insect-associated microorganisms.

*Chrysomya megacephala* (Fabricius, 1794) is a type of Diptera that is widely distributed globally and adopted for many functions. The *C. megacephala* larvae efficiently transform food waste, feces, and other organic wastes^8^. Adult *C. megacephala* are the main pollinators of rice, oil-seed rape, mango, and other crops^9^. In addition, *C. megacephala* is among the earliest insects to arrive at cadavers and is crucial for inferring the postmortem interval (PMI). However, the vector characteristics of *C. megacephala* limit its wide application. Studies have shown that *C. megacephala* harbors 12 times more bacteria than *Musca domestica*^10^. Recently, *Wohlfahrtiimonas chitiniclastica* was detected in *C. megacephala* collected at the Shanghai Pudong airport, causing fulminant sepsis in homeless patients^11^. There is an urgent need to implement the functional roles of *C. megacephala* and avoid the potential risks involved. However, research on its microbial community is incomprehensive, particularly at the pupal stage, which comprises approximately half of the entire larval development stage and is especially important entomological evidence in forensic medicine.

In this paper, the diversity and dynamics of the *C. megacephala* microbial community were studied during the pupal stage using third-generation sequencing technology. This research improves the understanding of microorganisms in *C. megacephala* and provides a basis for the prevention, control, and comprehensive utilization of *C. megacephala* in insect-microbe associations.

## Materials and methods

### Establishment of laboratory colony and sampling

*Chrysomya megacephala* was collected from a pig carcass placed in the Forensic Autopsy Centre of Suzhou, China (31° 21′ N, 120° 53′ E). The colony was raised in the laboratory for five years. At the beginning of the experiment, a culture dish containing fresh lean pork was placed in the insect-rearing cage to lay eggs. After oviposition, the dish containing the eggs was moved into a rearing box and placed in a microenvironment incubator LHP-300H (Yingmin Co., Ltd, Suzhou, China) set at 25 °C, 75% RH, and 12:12 (L:D) photoperiod. The larvae were observed every hour after feeding for pupation. After pupation, the pupae were moved into a new culture dish, and the pupation time was recorded. Development of the *C. megacephala* pupae takes approximately 5 days at 25 °C^12^, so the pupae were collected each day for 5 days, with three biological replicates for microbiome investigation, each of which used one whole pupa. After collection, the pupae were washed with 75% ethanol for 5 min and rinsed three times using sterile and ultrapure water for 1 min each time. The clean pupae were dried naturally at room temperature and stored at − 80 °C for subsequent experiments.

### DNA extraction, amplification, and sequencing

Total DNA was extracted from the whole pupa using a PowerSoil^®^ DNA Isolation Kit (MO BIO Laboratories, Carlsbad, CA, USA). DNA quality and quantity were measured using a Nanodrop 2000 (Thermo Fisher Scientific, Waltham, MA, USA).

The full-length 16S rDNA sequence was amplified using the universal PCR primers 27F (5′-AGRGTTTGATYNTGGCTCAG-3′) and 1492R (5′-TASGGHTACCTTGTTASGACTT-3′). Each PCR reaction contained 5 μL of KOD FX Neo Buffer (2×), 0.2 μl of KOD FX Neo, 0.3 μL of each primer (10 μM), 2 μL of dNTP, 10–100 ng of DNA, and topped with ddH_2_O to 10 μL. The PCR conditions involved the following: initial denaturation at 95 °C for 5 min, followed by 30 cycles of 95 °C for 30 s, 50 °C for 30 s, 72 °C for 1 min, and a final extension for 7 min at 72 °C.

PCR products were resolved on a 1.5% agarose gel and quantified with ImageJ software. High-quality amplicons from each sample were sequenced using the PacBio SMRT sequencing platform (Pacific Biosciences, Menlo Park, CA, USA) following the manufacturers’ instructions^13^.

### Data analysis

Raw reads were processed using the SMRT Link (Version 8.0) approach and the circular consensus sequencing (CSS) algorithm to generate reads with low error rates. Sequences of each sample were recognized according to barcodes using the Lima software (Version 1.7.0). The CSSs that contained primer sequences and met the length thresholds (1200–1650 bp) were reserved. Chimeric sequences were detected and removed using the UCHIME software (Version 8.0). Operational taxonomic units (OTUs) were clustered with 97% similarity using the USEARCH software (version 10.0). The taxonomic classification of each microbial OTU was assigned by RDP Classifier algorithm (Version 2.2) using the SILVA 16S rRNA database.

Rarefaction curves were generated with random sampling using the mothur software. The richness and diversity indices were evaluated on the QIIME2 platform^14^, and the differences in richness and diversity between the two groups were evaluated using a Student's *t*-test. The Spearman’s rank correlation coefficient determined the correlation between bacterial species, and the bacterial network was drawn using Python. Functional profiles were explored using the PICRUSt2 algorithm^15^. The Clusters of Orthologous Groups (COG) family information and Kyoto Encyclopedia of Genes and Genomes (KEGG) information of OTUs was obtained using the Greengene ID for each OTU. The description and function of each COG and KEGG were annotated based on the information on the COG and KEGG databases. The raw data generated in this study is accessible from NCBI, accession number PRJNA744036.

### Ethics approval

Ethics approval is not required because only insects were used in the study.

## Results

### The general profile of the sequencing data

**Figure 1:**
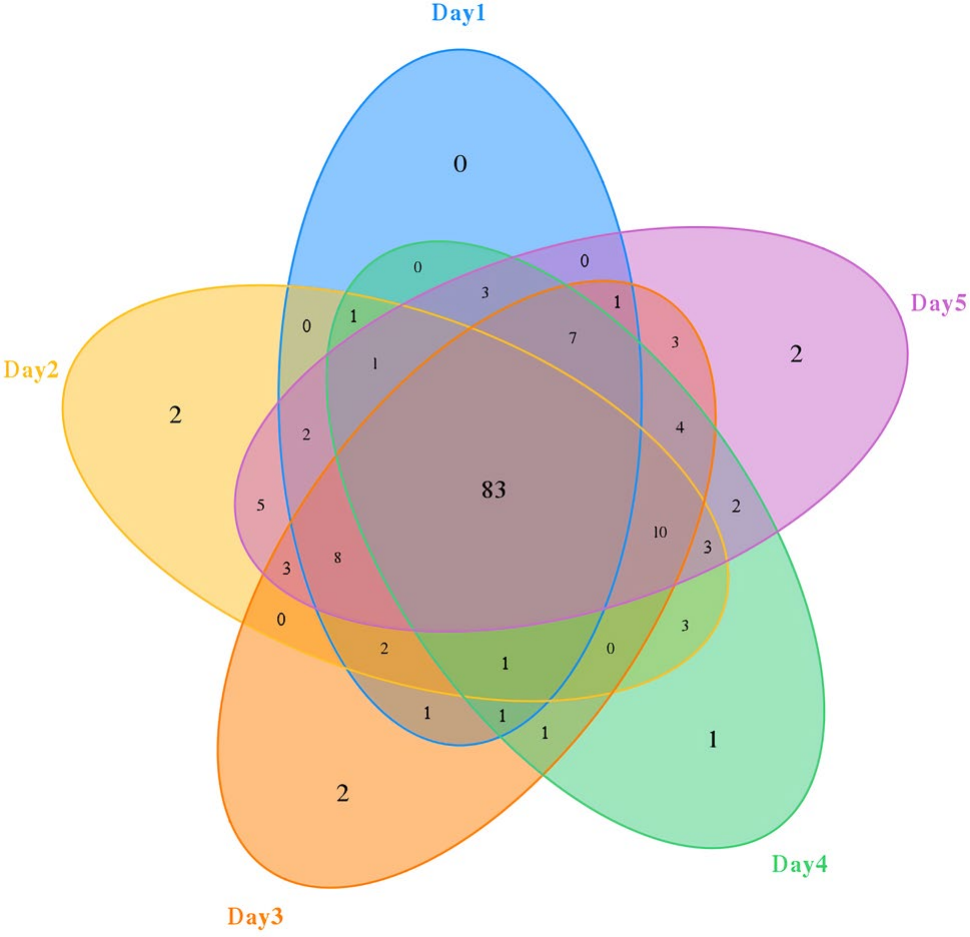
A Venn diagram of the bacterial OTUs in *Chrysomya megacephala* pupa at different developmental stages. Numbers within the compartments indicate OTU counts based on mathematical sets. OTU, operational taxonomic unit.

**Figure 2:**
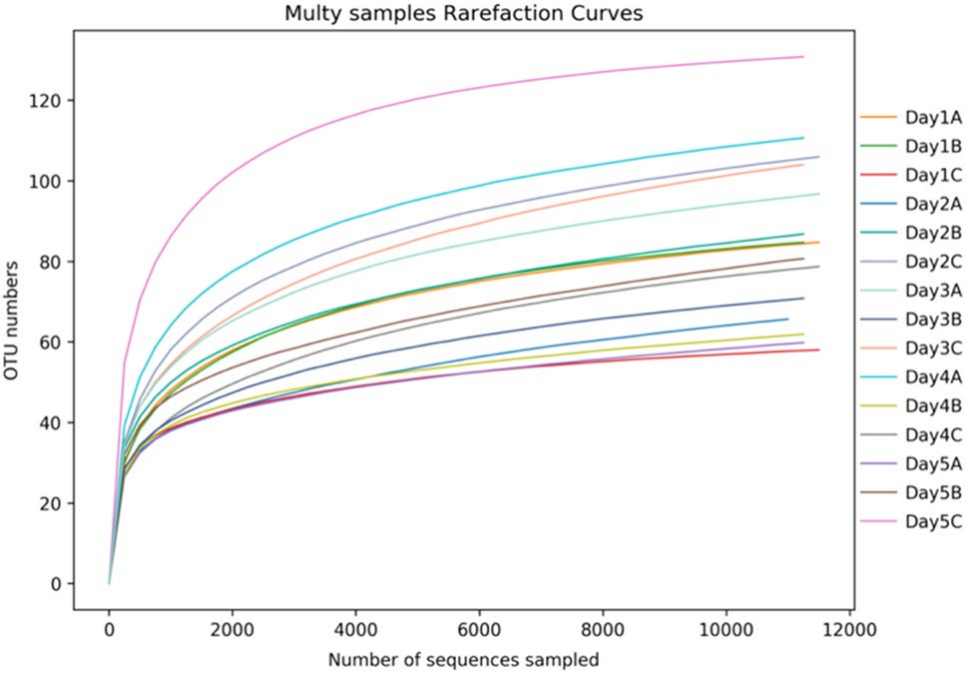
Rarefaction curves of bacterial communities in pupa samples of *Chrysomya megacephala*. (**A**–**C**) The different biological replicates.

The PacBio SMRT sequencing of the bacterial 16S rRNA amplicons from 15 samples yielded 194,651 raw CSSs. After quality filtering and chimera removal, 175,967 effective CCSs with 1451 bp average length were retained for further analyses. At 97% sequence similarity, 154 bacterial OTUs were obtained from all the samples. Among them, 83 bacterial OTUs were shared across all the samples, occupying 54% of the OTU repertoires (Fig. 1). The rarefaction curves of all samples almost reached the saturation plateau (Fig. 2) and ~ 99% Good's coverage for each sample, indicating sufficient sequencing depth and coverage of bacterial diversity.

### Microbiota taxonomical composition

**Figure 3:**
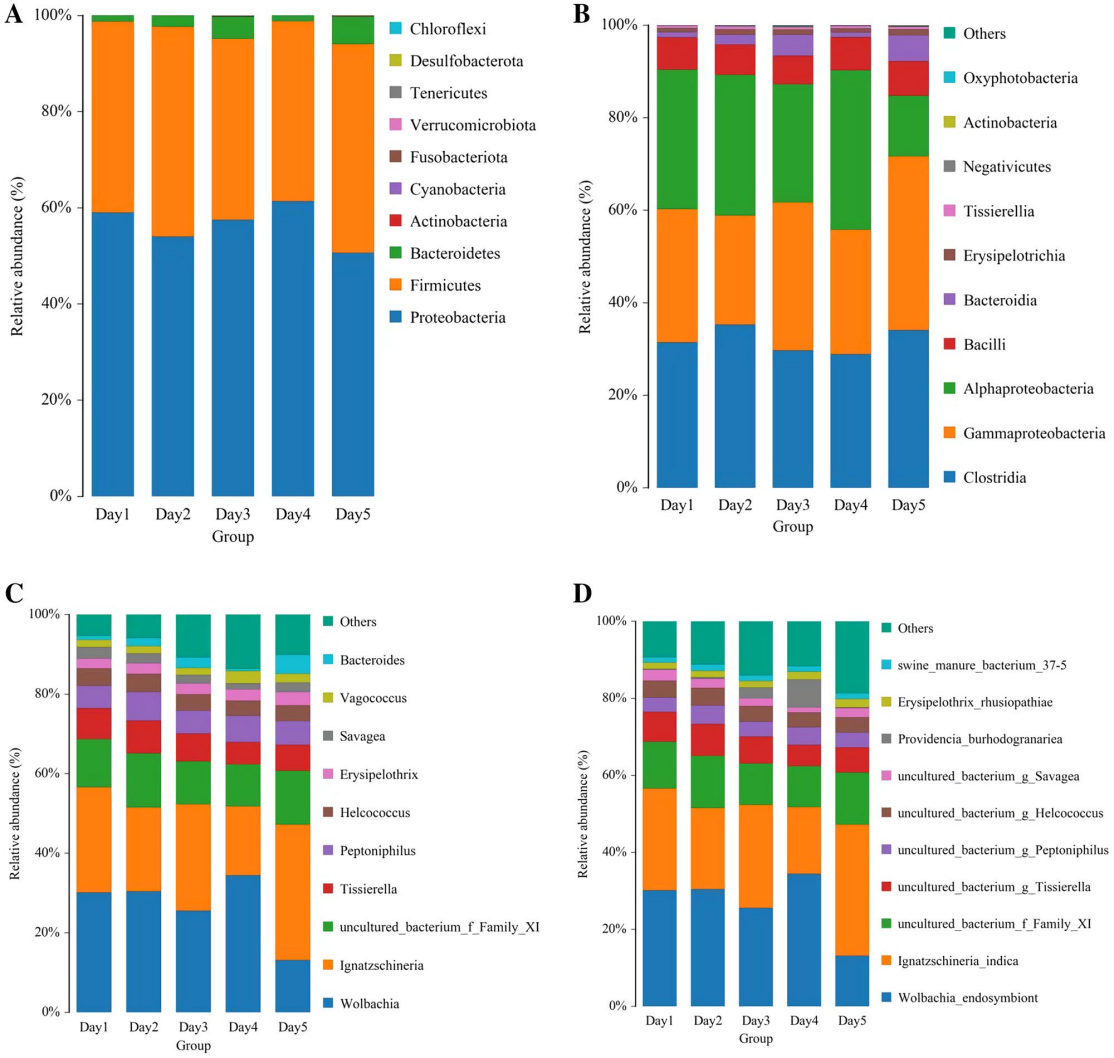
The top 10 relative abundance of bacteria during the *Chrysomya megacephala* pupal stage at the levels for phylum (**A**), class (**B**), genus (**C**), and species (**D**).

The bacterial OTUs in all the samples were annotated into 10 phyla, 15 classes, 28 orders, 50 families, 88 genera, and 130 species. The relative abundance of the top 10 from each phylum, class, genus, and species is shown in the species distribution histogram at different taxonomic levels. The remaining species are combined into OTHERS (Fig. 3). Proteobacteria was the most predominant phylum, accounting for more than 50% in each group. During the *C. megacephala* pupal stage, Clostridia, Gammaproteobacteria, and Alphaproteobacteria were the three most dominant bacterial communities, followed by Bacilli and Bacteroidia. *Wolbachia* and *Ignatzschineria* had over 50% relative abundance at the genus level, but the relative abundance of *Wolbachia* in the Day5 group was lower than those of the other groups. Similarly, *Wolbachia endosymbiont* and *Ignatzschineria indica* were the two predominant species in the bacterial communities accounting for > 50% of each group, and the relative abundance of *Wolbachia endosymbiont* in the Day5 group was lower than that of the other groups. In addition, *Erysipelothrix rhusiopathiae*, the etiological agent of swine erysipelas, was identified in the bacterial communities of *C. megacephala*, probably obtained by feeding in the larval stage.

**Figure 4:**
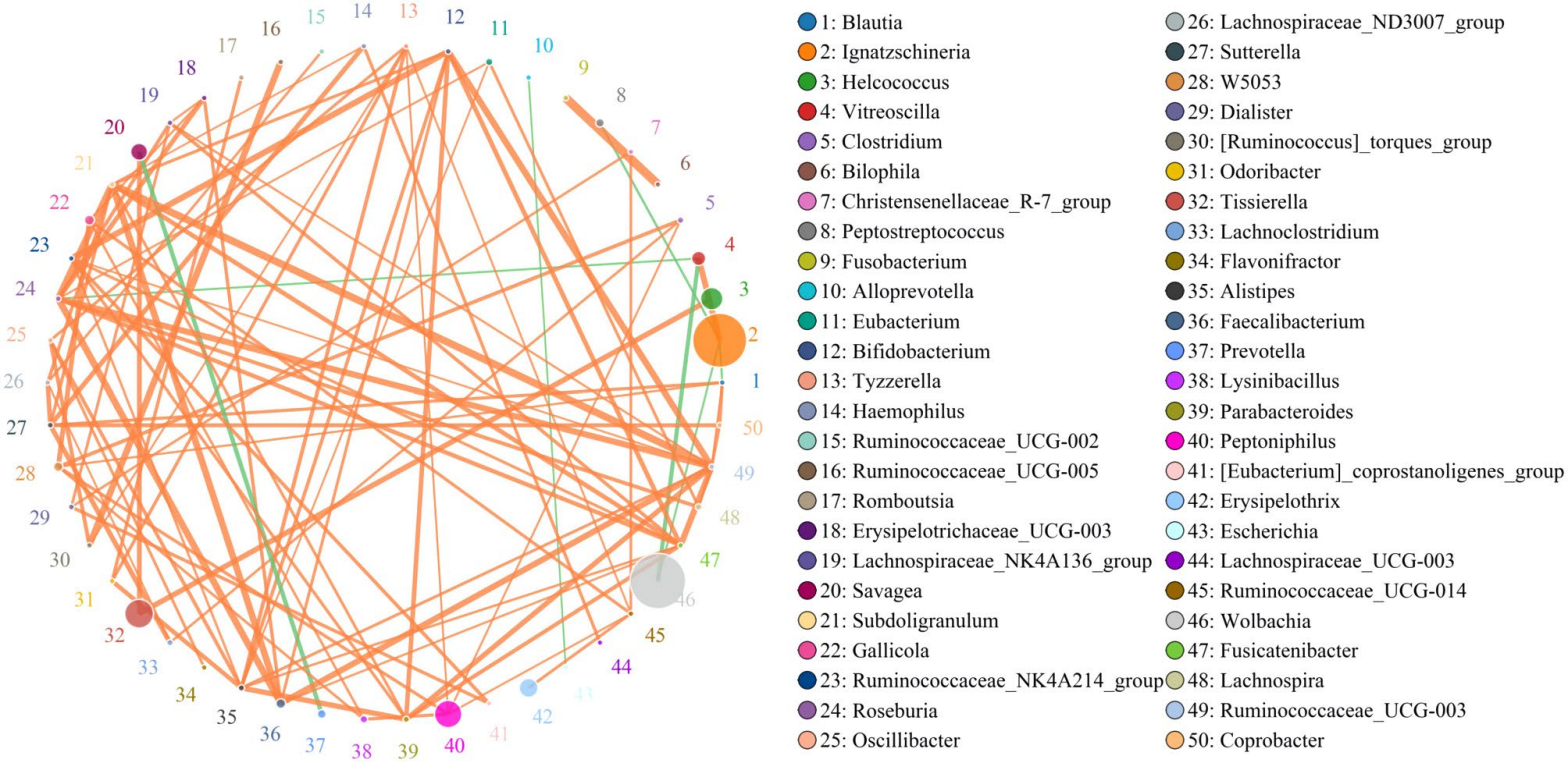
Bacterial networks at the genus level. Orange and green lines represent positive and negative correlations, respectively.

Bacterial networks of the top 50 genera with the highest correlation (Fig. 4). The Spearman’s rank correlation between *Wolbachia* and *Ignatzschineria* was negative, suggesting a competitive relationship between the two genera.

### Comparison of the bacterial population

The richness and diversity of the bacterial communities in different developmental stages of *C. megacephala* pupa were estimated using the ACE, Chao1, Simpson, and Shannon indices (Table 1). The number of bacterial OTUs increased, but there was no significant difference between the groups (P > 0.05). The richness and diversity of bacterial communities among the five groups were not significantly different between groups. The ACE and Chao1 indices indicated that the Day5 group exhibited the highest species richness. The higher values of Shannon and Simpson diversity indices suggested that the taxonomic diversity of the Day5 group was not lower than other groups in contrast to other developmental stages.

**Table 1:** Richness and diversity estimates of the 16S rRNA gene libraries from the sequencing analysis.

Sample	OTUs	ACE	Chao1	Simpson	Shannon	Coverage
Day 1	111	84.78	82.23	0.80	3.36	0.99
Day 2	126	115.61	108.30	0.82	3.57	0.99
Day 3	129	108.69	108.65	0.78	3.49	0.99
Day 4	121	102.56	100.09	0.80	3.44	0.99
Day 5	139	116.84	109.31	0.82	3.81	0.99

### Functional prediction

**Figure 5:**
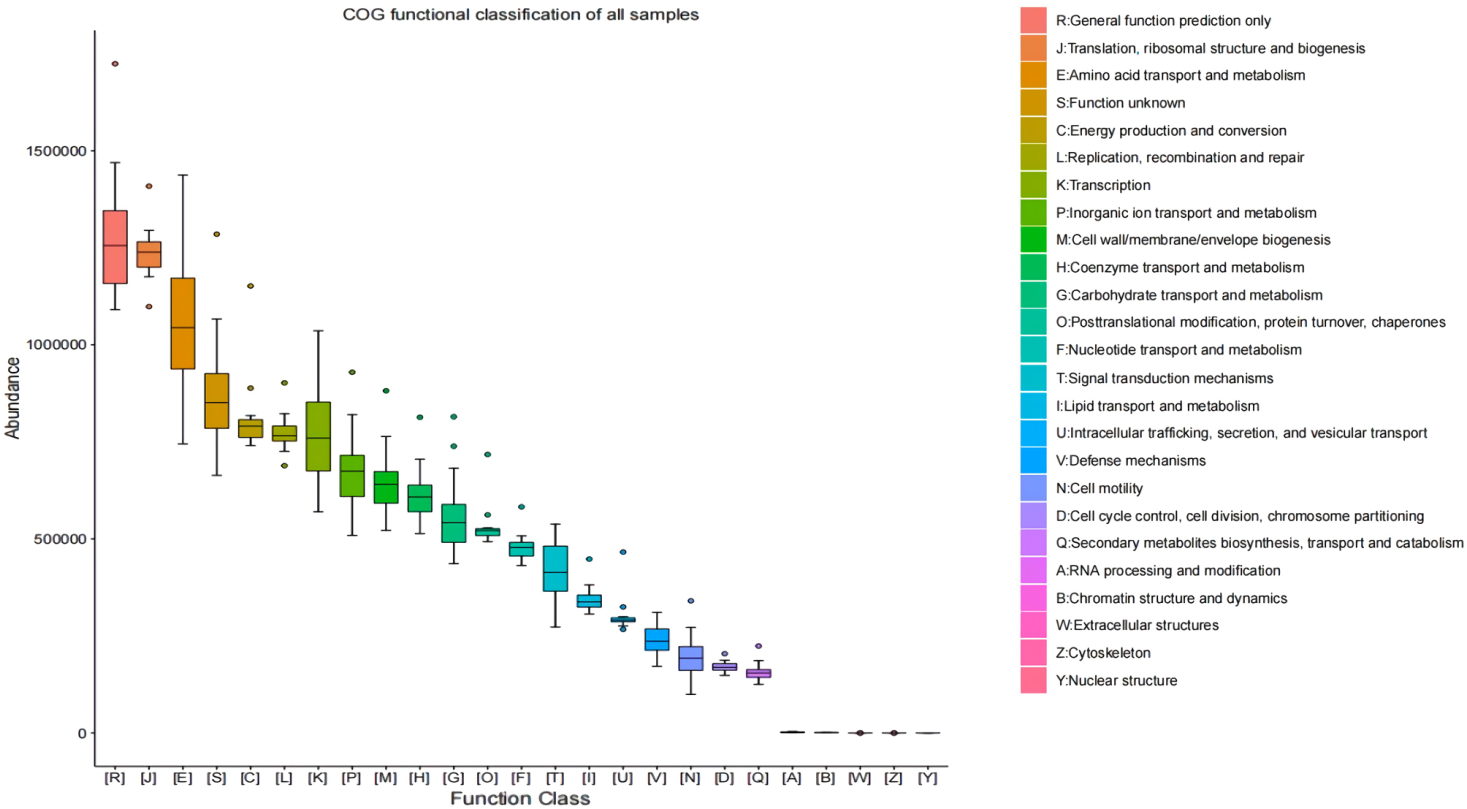
COG functional secondary classification of all the samples. Different colors represent the various functions.

A total of 24 Level 2 COG functions were annotated except the nuclear structure (Y), and three functions had median abundances higher than 1,000,000 (Fig. 5). In addition, translation, ribosomal structure and biogenesis (J), and amino acid transport and metabolism (E) had the highest predicted abundances. Moreover, RNA processing and modification (A), Chromatin structure and dynamics (B), Extracellular structures (W), and Cytoskeleton (Z) had the lowest predicted abundance.

**Figure 6:**
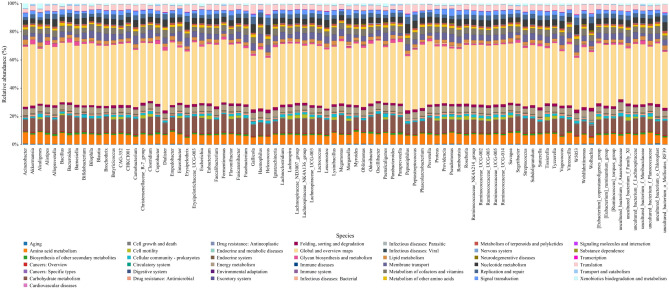
KEGG functional secondary classification at the genus level. The X-axis indicates the species, and the Y-axis shows the relative abundance percentage of metabolic pathways.

In the KEGG functional annotation, 43 Level 2 KEGG pathways were predicted for all the samples (Fig. 6). The KEGG bacterial metabolic pathways are classified into global and overview maps for amino acid metabolism, carbohydrate metabolism, replication and repair, energy metabolism, metabolism of cofactors and vitamins, translation, membrane transport, and nucleotide metabolism. Moreover, 256 Level 3 KEGG pathways were predicted for all the samples (Supplementary Table S1). Several Level 3 pathways are related to drug resistance, including cationic antimicrobial peptide resistance, ß-lactam resistance and vancomycin resistance, providing a reference method to control *C. megacephala*.

## Discussion

The microbiomes associated with insects are important in mediating host health and fitness. In recent years, numerous studies have explored the microbial diversity and variations across different developmental stages in insects, particularly for pests, including *Bactrocera dorsalis*^16^, *Monochamus alternatus*^17^, and *Zeugodacus tau*^18^. Previously, bacterial communities were investigated using inefficient, low-throughput culture-based or conventional molecular methods^19,20^, inevitably underestimating the microbial abundance. The advancements in sequencing technology have inspired more research on insect microbial communities, thereby enriching the information on the microbiome of insects. However, a comprehensive understanding of the *C. megacephala* pupal stage microbiome remains unclear. Therefore, this paper presents a study of the diversity and dynamics of bacteria in the pupal stage of *C. megacephala* using third-generation sequencing of bacterial 16S rRNA. The results provide a better understanding of the *C. megacephala* microbiome.

This annotation results demonstrate that the bacteria in the pupal stage of *C. megacephala* are rich and diverse, but the diversity is indiscrete. At the phylum level, Proteobacteria, Firmicutes, and Bacteroidetes were the three predominant phyla, similar to the observation from the housefly *Musca domestica*^21^, possibly owing to a semblable ecological niche. The bacterial community analysis identified Clostridia and Gammaproteobacteria as the two predominant bacterial classes in the pupal stage of *C. megacephala* with ~ 30% relative abundances. However, another study of the gut bacteria across the lifecycle of *C. megacephala* showed Gammaproteobacteria as the dominant class with over 60% relative abundance. These results suggest that Clostridia may be from other *C. megacephala* tissues apart from the gut.

Compared with the previous results about *C. megacephala* bacterial communities that were determined using culture-based or conventional molecular methods, the microbial diversity was much higher in this study using third-generation sequencing technology^22^. However, we cannot identify some bacteria to the species level, such as *Klebsiella pneumoniae* and *Aeromonas hydrophila*^23^, so culture-based and conventional molecular methods are also important.

*Ignatzschineria indica* and *Wolbachia endosymbiont* were the two predominant species in the bacterial communities in the *C. megacephala* pupal stage. *Ignatzschineria indica* is a Gram-negative bacterium commonly associated with maggot infestation and myiasis, a probable marker for myiasis diagnosis^24,25^. *Wolbachia* are intracellular symbiotic bacteria widely distributed in the reproductive tissues of arthropods. They cause reproductive alterations in their hosts, such as cytoplasmic incompatibility (CI)^26^, feminization^27^, killing males^28^, and inducing parthenogenesis (PI)^29^. *Wolbachia* increases the resistance to arbovirus infection, resulting in decreased virus transmission. The reproductive regulation of *Wolbachia* on target organisms may be important in future biological prevention and pest control. Since *Wolbachia* causes CI, *Wolbachia*-infected populations can be established and released to reduce to the environment to reduce the reproductive potential of harmful target insect populations. Modified *Wolbachia* that harbor anti-parasitic or anti-viral genes can be adopted to control virus transmission in insects carrying viruses^30^.

However, few studies have reported that *Ignatzschineria* and *Wolbachia* can coexist in an individual insect, despite their status as common bacterial genera. Several possibilities may explain this analytical discrepancy. Firstly, in this study, Spearman’s rank correlation between *Wolbachia* and *Ignatzschineria* showed a negative correlation, suggesting a competitive relationship between *Wolbachia* and *Ignatzschineria*. Secondly, the previous investigations of bacterial communities applied inefficient, low throughput culture-based or conventional molecular methods, potentially generating incomplete results. Finally, numerous studies have established that microbial communities differ between insect populations because of different sampling techniques and procedures^31^. This study analyzed *C. megacephala* sampled from a laboratory population reared with pork for five years. Nevertheless, the significant decrease in the relative abundance of *Wolbachia* observed at the end of the pupal development is unsolved, thus, required further studies.

Traditionally, the most common method for pest control is by chemical pesticides. However, the excessive use of chemical pesticides causes the rapid build-up of pesticide resistance and environmental pollution. Therefore, it is urgent to develop biological control methods for pests. *Nasonia vitripennis* (Walker), is an important parasitoid whose female wasp stings, injects venom, and lays eggs in different fly pupae, where parasitoid eggs, larvae, pupae, and early-stage adults develop. *N. vitripennis* lives in species of the family Calliphoridae, Sarcophagidae, and Muscidae, where their larvae feed on fly pupae, allowing *N. vitripennis* to function as a biological agent to control the flies.

The microbial communities of fly species and *N. vitripennis* live in an enclosed environment, providing more opportunities for the *N. vitripennis*-fly communication. Therefore, the impacts of micro-communities of the fly hosts on *N. vitripennis* are worth studying, precisely at the pupal stage. Studies of different fly hosts and their corresponding *N. vitripennis* showed diverse core microbiota, and so other fly hosts shaped the bacterial diversity of their parasitic wasps^32^. In addition, parasitic wasps infected with *Wolbachia* produced more female offspring than uninfected ones, further emphasizing the need to improve biological prevention and control efficiency^33^. Therefore, a deliberate focus to study the micro-communities of different fly species at the pupal stage and the interaction between the fly species and *N. vitripennis* will guide the development and utilization of *N. vitripennis* as biological agents for the prevention and control of flies.

Approximately half of the bacteria identified at the species level in this study are pathogens or conditional pathogens (Supplementary Table S2), *Escherichia coli*, *Providencia burhodogranariea,* and *Morganella morganii,* among others. Another uncommon pathogenic bacterium, *Erysipelothrix rhusiopathiae* was also identified at the species level. *E. rhusiopathiae* is the etiological agent of swine erysipelas and causes economically important chicken, duck, and sheep diseases. Although *E. rhusiopathiae* primarily infects pigs, it also infects various domestic and wild mammals, including marine mammals, birds, and humans. Humans infected with *E. rhusiopathiae* develop large areas of red spots on their body. Severe *E. rhusiopathiae* infection causes endocarditis and septicemia, which have a 38% mortality rate^34^.

However, very few studies have focused on the insects that transmit *E. rhusiopathiae*^35^. Considering that the *C. megacephala* samples in this study were obtained from a laboratory population reared for five years, it is likely that the *E. rhusiopathiae* originated from infected pork and were transmitted to *C. megacephala* through feeding. Thus, disease-vector insects can infect and spread pathogens beyond their feeding activities, and disease-vector insects require more comprehensive prevention and control methods (“Supplementary information”).

In conclusion, this study comprehensively investigated the pupal stage microbiome of *C. megacephala* using third-generation sequencing to deepen the understanding of *C. megacephala* microbial communities on the whole. The study provides a basis for subsequent studies of biological control and the comprehensive utilization of *C. megacephala*. Future studies should focus on the transmission patterns and biological functions of these microbial species.

## Supplementary Information

Supplementary Table S1.

Supplementary Table S2.
